# Adolescent Epstein-Barr Virus Hepatitis Without Typical Mononucleosis Symptoms and Gilbert’s Syndrome: A Case Report

**DOI:** 10.7759/cureus.71925

**Published:** 2024-10-20

**Authors:** Roy L Dennis, Dean Hasan, Krista Jackson, Caylyne Arnold

**Affiliations:** 1 Internal Medicine, Grand Strand Medical Center, Myrtle Beach, USA; 2 Internal Medicine, Edward Via College of Osteopathic Medicine-Carolinas Campus, Spartanburg, USA; 3 Emergency Medicine, Grand Strand Medical Center, Myrtle Beach, USA

**Keywords:** epstein-barr virus, fever with jaundice, gilbert’s syndrome, hepatitis, unconjugated hyperbilirubinemia

## Abstract

Epstein-Barr virus (EBV) is one of the most common infections worldwide that presents with a multitude of symptoms such as lymphadenopathy, fever, and malaise and has associations with Hodgkin's lymphoma. EBV can cause elevations in transaminase values and hyperbilirubinemia; however, EBV will rarely cause hepatitis with cholestatic features. Here we report a case of a 15-year-old male with a past medical history of potential Gilbert’s syndrome who presented with jaundice, scleral icterus, mild abdominal pain, and low-grade fever. Further work-up revealed elevated transaminase values, abnormal coagulation studies, and thrombocytopenia. The patient was monitored and diagnosed with EBV hepatitis, then treated with supportive therapy and IV vitamin K for the resolution of abnormal coagulopathy. The following report presents a patient with EBV hepatitis without the typical presentation of EBV, with a process for diagnosing and managing patients.

## Introduction

Epstein-Barr virus (EBV), a member of the herpesvirus family, is primarily known for causing infectious mononucleosis, often referred to as "mono" or the "kissing disease." While EBV is commonly associated with symptoms such as fever, sore throat, and lymphadenopathy, it can also manifest in less typical ways, including hepatitis [1]. Hepatitis refers to inflammation of the liver, which can be caused by various infectious agents and conditions.

EBV-induced hepatitis is relatively rare compared to these more common forms. It typically occurs in the context of infectious mononucleosis, but isolated EBV hepatitis without the classic symptoms of mononucleosis is uncommon [2]. When EBV hepatitis does occur, it often presents with mild liver enzyme elevations and jaundice, but without the pronounced features of mononucleosis [3]. 

A key challenge in diagnosing EBV hepatitis is the variability in mononucleosis test results. Anti-VCA IgM serology is the most reliable diagnostic test for EBV; however, serology studies are usually slow and expensive. Monospot testing using equine RBCs to detect EBV heterophile antibodies is often utilized for its rapid results. However, within the first week of symptom onset, monospot testing has a 25% false negative [4]. This can delay the recognition of EBV as the underlying cause of hepatitis, especially in cases where the clinical presentation is atypical.

In cases of EBV hepatitis, laboratory findings often reflect mild hepatocellular injury. For example, elevated aminotransferases typically remain less than five times the normal levels, and bilirubin levels may rise, though rarely exceeding significant thresholds [5]. As illustrated by our case, the patient had a total bilirubin level of 9.5 mg/dL, with indirect bilirubin constituting 9.4 mg/dL, and a prothrombin time (PT) of 14.7 seconds with an international normalized ratio (INR) of 1.31. Platelet counts may also be reduced, as evidenced by their count of 122 x 10^9/L. Although such elevations can be linked to intrahepatic cholestasis or hemolytic anemia, they are often subtle and can resolve spontaneously without specific treatment [5]. 

Another important condition to consider in the differential diagnosis of hepatitis with jaundice in this case is a past medical history of Gilbert’s syndrome. Gilbert’s syndrome is a benign genetic condition that results in elevated levels of bilirubin due to reduced activity of the enzyme UDP-glucuronosyltransferase [6]. This condition is typically asymptomatic and does not require specific treatment. It is often identified incidentally during workup for other conditions. 

This case report presents a patient with a rare cause of hepatitis due to EBV, however, an assumed prior history of Gilbert's syndrome with lab value abnormalities made the diagnosis more difficult. The case underscores a thorough workup is crucial when diagnosing hepatitis, especially when the presentation is atypical. Diagnostic investigations generally include blood tests to assess liver function, imaging studies to evaluate liver structure, and possibly liver biopsy in certain cases. Gilbert’s syndrome, while benign, should be distinguished from other liver pathologies to avoid unnecessary concern and treatment because it can be exacerbated by viral causes.

## Case presentation

Our patient was a 15-year-old male with a past medical history of neonatal physiologic jaundice, who presented to the emergency department with a new onset of jaundice. Prior to the onset of jaundice, the patient endorsed systemic symptoms of a fever (temp max 103.9 ℉), headache, and malaise for approximately three days. Also, the patient had decreased appetite from abdominal fullness and dark orange-brown-colored urine without dysuria or hematuria. We discussed with the patient's parents a history of having Gilbert's syndrome at birth. However, the parents never had a full diagnostic workup after the patient was treated at birth. As a neonate, the patient had unconjugated hyperbilirubinemia and jaundice, and their primary care provider made the clinical diagnosis of Gilbert’s syndrome. Gilbert’s syndrome does not require further treatment or increase risks for other pathologies, so no other diagnostic studies were done during the patient's childhood. Otherwise, a review of the systems was negative for chest pain, dyspnea, sore throat, vomiting, congestion, and changes in color in the stool. 

**Figure 1:**
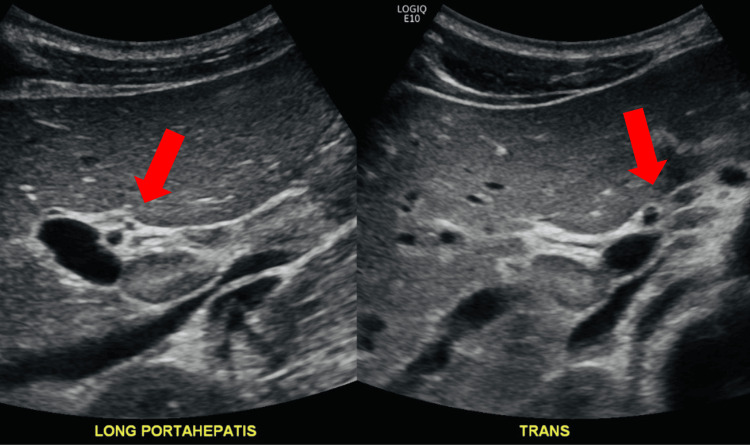
RUQ ultrasound with porta hepatitis lymph nodes RUQ: right upper quadrant

**Figure 2:**
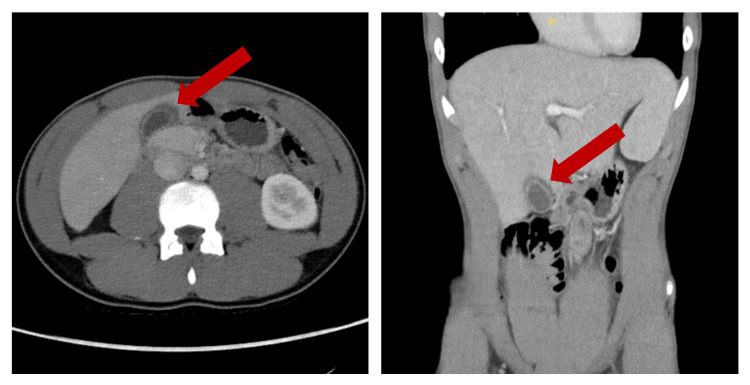
Initial CT abdomen and pelvis with nonspecific pericholecystic fluid

Upon examination, the patient’s vital signs were relatively normal, with a blood pressure of 134/78, a temperature of 97.9, and saturating at 99% O2 on room air. Physical examination revealed bilateral scleral icterus with jaundice and right upper quadrant tenderness without palpable hepatomegaly or splenomegaly. Otherwise, the patient appeared to be a well-developed, well-nourished male in no acute distress. The remaining ENT, cardiac, pulmonary, and skin examinations were normal. A CT scan of the abdomen and pelvis with contrast revealed nonspecific mild pericholecystic fluid, a right upper quadrant ultrasound revealed borderline porta hepatis lymph nodes and urine analysis was unable to be read due to color interference (Figures 1, 2). 

Upon evaluation, the patient was a non-toxic appearing adolescent in no acute distress with abdominal pain, scleral icterus, and orange-colored dark urine. There was a differential diagnosis of hyperbilirubinemia, hepatitis, cholecystitis, mononucleosis, or strep throat since there was right upper quadrant discomfort and jaundice. The patient had a negative strep, flu, and COVID-19 test. A complete blood count (CBC) and comprehensive metabolic panel (CMP) were obtained along with viral studies to rule out the causes of the patient's presenting symptoms. The patient had no leukocytosis, anemia, or electrolyte abnormalities, and had a normal lipase range with an undetectable acetaminophen level. However, the CMP had multiple abnormalities, but a negative hepatitis panel and negative infectious mononucleosis assay (Table 1).

**Table 1:** Initial laboratory values on presentation to the emergency department

Variables	Patient’s values	Normal range
White blood cell	4.9 K/mm3	3.7-10.1 K/mm3
Red blood cell	4.47 M/mm3	4.55-5.47 M/mm3
Hemoglobin	14.2 gm/dL	14.0-16.4 gm/dL
Hematocrit	41.0 %	40.0-47.2 %
Mean corpuscular volume	91.8 fL	81.8-94.6 fL
Mean corpuscular hemoglobin	31.7 pg	27.9-33.1 pg
Mean corpuscular hemoglobin concentration	34.5 g/dL	33.4-35.6 g/dL
Red blood cell distribution width	13.2 %	11.6-14.0 %
Platelets	122 K/mm3	150-400 K/mm3
Neutrophil %	26.4 %	40.1-81.3 %
Neutrophil #	1.3 K/mm3	1.4-6.5 K/mm3
Lymphocyte %	54.1 %	17.0-47.0 %
Lymphocyte #	2.7 K/mm3	1.2-3.4 K/mm3
Monocyte %	16.2 %	5.5-11.9 %
Monocyte #	0.8 K/mm3	0.1-0.6 K/mm3
Eosinophil %	2.2 %	0.0-5.5 %
Eosinophil #	0.1 K/mm3	0.0-0.7 K/mm3
Basophil %	1.1 %	0.0-1.1 %
Basophil #	0.1 K/mm3	0.0-0.2 K/mm3
Sodium	137 mmol/L	137-145 mmol/L
Potassium	4.2 mmol/L	3.5-5.1 mmol/L
Chloride	100 mmol/L	96-107 mmol/L
Carbon Dioxide	33 mmol/L	22-32 mmol/L
Anion Gap	4.0 mEq/L	3-11 mEq/L
BUN	11 mg/dL	7-20 mg/dL
Creatinine	1.00 mg/dL	0.7-1.5 mg/dL
Blood urea nitrogen/creatinine	11	10-20
Glucose	98 mg/dL	74-106 mg/dL
Calcium	9.2 mg/dL	8.4-10.2 mg/dL
Magnesium	2.1 mg/dL	1.6-2.3 mg/dL
Total bilirubin	9.5 mg/dL	0.1-1.1 mg/dL
Direct bilirubin	0.1 mg/dL	0.0-0.2 mg/dL
Indirect bilirubin	9.4 mg/dL	0.2-0.8 mg/dL
Aspartate aminotransferase	104 U/L	15-46 Units/L
Alanine aminotransferase	130 U/L	13-69 Units/L
Alkaline phosphatase	190 U/L	74-390 Units/L
Creatine kinase	64 U/L	30-170 Units/L
Total protein	7.6 gm/dL	6.3-8.2 gm/dL
Albumin	4.4 gm/dL	3.5-5.0 gm/dL
Globulin	3.3 gm/dL	2.5-4.5 gm/dL
Lipase	58 U/L	23-300 Units/L
Prothrombin time	14.7 seconds	9.9-13.6 seconds
Activated partial thromboplastin time	30.5 seconds	26.2-37.2 seconds
International normalized ratio	1.31	0.88-1.2
Hepatitis A IgM antibody	Negative	Negative
Hepatitis B surface antigen	Negative	Negative
Hepatitis B core IgM antibody	Negative	Negative
Hepatitis C antibody	Negative	Negative
Infectious mono qualitative assay	Negative	Negative
Group A strep rapid	Negative	Negative
Acetaminophen level	<10.0 mcg/mL	10.0-30.0 mcg/mL

The patient was initially treated with 1 L fluid bolus and 15 mg ketorolac IV while awaiting consultation with the pediatric hospitalist. Upon discussion with the pediatric hospitalist, the patient was transferred to a medical center with a pediatric gastroenterologist (GI) specialist due to the elevated unconjugated hyperbilirubinemia and prolonged coagulation studies.

Upon arrival at the tertiary care center with pediatric gastroenterology, the patient had normal vitals with a heart rate of 83 BPM, temperature of 98.6 F, and blood pressure of 131/66. A physical exam revealed diffuse jaundice, scleral icterus, and pain with palpation in the right upper quadrant and epigastric region with deep inhalation. Otherwise, the patient appeared to be a well-developed, well-nourished male with normoactive bowel sounds. Initial lab values upon arrival still revealed acute abnormalities and prolonged coagulation (Table 2).

**Table 2:** Laboratory values upon admission to tertiary pediatric facility

Variables	Patient’s values	Normal range
Total bilirubin	13.5 mg/dL	0.2-1.2 mg/dL
Aspartate aminotransferase	88 U/L	14-35 Units/L
Alanine aminotransferase	104 U/L	9-24 Units/L
Prothrombin time	14.6 seconds	9.9-13.6 seconds
International normalized ratio	1.07	0.90-1.2
Gamma-glutamyl transferase	180.0 U/L	7.0-21.0 Units/L
C-Reactive protein	2.260 mg/dL	0.000-1.000 mg/dL
Epstein-Barr virus DNA blood viral load	1,607 IU/mL	Not detected
Neutrophils %	39%	50-75%
Monocytes %	17%	0-10%
Reactive lymphocytes %	17%	0-4%

After being admitted, the patient's labs were monitored overnight, and he was given IV fluids as needed until pediatric GI consultation. The pediatric gastroenterologist recommended a repeat right upper quadrant ultrasound and repeat lab values. 

**Figure 3:**
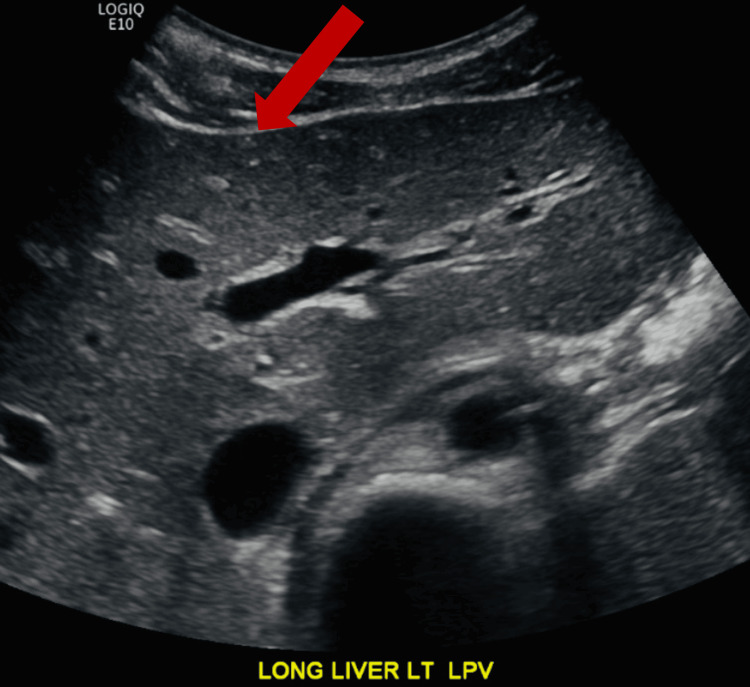
Starry sky appearance of the liver

Abdominal ultrasound resulted in hepatosplenomegaly with gallbladder wall thickening, periportal lymphadenopathy, and starry sky appearance on the liver parenchyma, which suggested hepatitis (Figure 3). Also, lab results consistently returning positive for EBV polymerase chain reaction (PCR) led to the diagnosis of EBV hepatitis.

The patient was then treated for one more day with oral intake fluids (PO) and supportive treatment with repeated lab checks. The patient’s coagulation profile normalized two days after IV administration of vitamin K 2.5 mg. After the coagulation profile normalized, the patient was discharged with down-trending bilirubin levels and transaminase lab values. Further diagnostic outpatient workup for Gilbert’s syndrome and follow-up labs were discussed to be done a few weeks after discharge.

## Discussion

In clinical practice, when presented with jaundice, it’s important to keep a broad differential and to rule out causes of jaundice including medication-induced, viral source, or hereditary hyperbilirubinemias. The most common cause of acute jaundice worldwide is hepatitis A; however, a thorough patient history is imperative to rule out common causes. When patients present with conjugated bilirubin levels > 3 mg/dL, uncommon causes of jaundice should be considered such as Epstein-Barr virus. EBV will present with systemic symptoms of fever, malaise, atypical lymphocytosis, and jaundice [1,3-5].

In retrospective studies, it was observed the most common ages for EBV-induced hepatitis are in the age group of 4-8 years and 14-18 years. However, the trend is more common in the age groups of 14-18 years. The pathogenetic mechanism of EBV-induced hepatitis is still misunderstood, although many hypotheses have been proposed. The theory is that EBV leads to an increase in cytokines and inflammation, causing autoimmune oxidative damage to the liver parenchyma [6,7]. 

Gilbert’s syndrome is a benign condition that can cause an increase in unconjugated hyperbilirubinemia when under stress-induced conditions. The most common presentation of Gilbert’s syndrome is jaundice which occurs when the body is under physiological stress like illness, physical exertion, dehydration, or skipping meals [6]. The patient in this case was thought to have a prior history of Gilbert’s syndrome, which made diagnosing their condition of EBV hepatitis difficult due to overlapping symptoms between the conditions. Both conditions present with jaundice; however, EBV hepatitis will have fever, malaise, and atypical lymphocytosis. Also, Gilbert’s syndrome will have an elevated total bilirubin of 3.5 or less, while EBV hepatitis can have substantially larger elevations in bilirubin. Also, Gilbert’s syndrome should not induce prolongation of the clotting cascade or cause increased transaminase levels. In this case, the diagnosis was difficult because the patient presented a non-toxic appearance and was afebrile while still having elevated bilirubin with jaundice and scleral icterus with a prolonged clotting cascade and increased transaminase levels. When a patient presents with jaundice and elevated bilirubin it is important to keep a broad differential and get a thorough history to determine if the patient is having a physiological response from stress from their prior history of Gilbert’s, compared to other viral etiologies.

Diagnostic studies may present challenges, as in this case with the patient having a negative infectious mononucleosis assay. The original infectious mononucleosis assay test has a specificity of 91% and a sensitivity of 87%; however, it is insensitive during the first few weeks of illness [5]. While EBV-specific antibody serology has a higher sensitivity of 97% and specificity of 94% than the infectious mononucleosis assay [8]. As in this case, if the mononucleosis assay is negative, but there is still a strong suspicion for EBV, then viral serologies should be checked more than once, especially if the patient is not showing improvement with supportive treatments for jaundice. Another interesting aspect of this case presentation is the elevated transaminase without the typical symptoms of EBV: tonsillitis, lymphadenopathy, nausea, vomiting, splenomegaly, or maculopapular generalized rash. The development of jaundice and elevated transaminase values occurs in less than 10% of case presentations of EBV [8-12]. Although this type of presentation could be considered rare for EBV, it's imperative to have a wide differential for EBV in adolescents presenting with these symptoms.

Thrombocytopenia is when platelet levels drop below 150,000 platelets per microliter of circulating blood. Patients with EBV may present with mild thrombocytopenia and atypical lymphocytosis; however, the features of jaundice with those symptoms in this case made the presentation challenging to diagnose [8,12]. When presented with these symptoms and lab abnormalities in an adolescent, it is important to consider consultation with a pediatric gastroenterologist to ensure appropriate diagnosis and management. Additional, potentially life-threatening etiologies of thrombocytopenia, prolonged coagulation cascade, and jaundice include autoimmune hemolysis, glucose-6-phosphate dehydrogenase deficiency, red blood cell membrane defects, and hemoglobin disorders. A thorough history and diagnostic testing are required to rule out these conditions and to prevent life-threatening complications. 

In this case, management consisted of oral rehydration and IV vitamin K to treat the prolonged coagulopathies; however, oral or IV vitamin K may be used to treat the prolonged coagulopathy. Vitamin K has been shown to decrease elevated INR or decrease prolonged PT as seen in the patient in this case. Symptomatic treatment for patients varies depending on symptom presentation. There have been cases when IV N-acetylcysteine has been used in the management of EBV hepatitis to improve liver enzymes. Further studies are indicated for the use of IV N-acetylcysteine in conjunction with supportive therapies to treat EBV-induced hepatitis [2].

## Conclusions

The patient was found to have hepatitis caused by EBV, presenting with jaundice, abdominal discomfort, and fever. This patient's presentation, with elevated bilirubin, transaminase values, and coagulation factors, is considered an abnormal presentation of EBV in pediatrics. When presented with patients with unconjugated hyperbilirubinemia greater than 3.5 mg/dL, jaundice, and abnormal lab values, it is imperative to have a broad differential for potential causes of cholestatic hepatitis.

A comprehensive diagnostic workup and thorough past medical history are imperative for diagnosing EBV hepatitis. Even if a monospot test is initially negative, it is important to follow up with EBV serology testing, since early presentation of acute EBV-induced hepatitis does not necessarily present positive, so lab values should be trended over days to weeks if symptoms persist. Management for patients with this presentation is IV vitamin K for coagulopathy abnormalities, supportive therapy with PO or IV fluids, and persistent monitoring of lab values with follow-up weeks to months later.
